# Proactive community case management decreased malaria prevalence in rural Madagascar: results from a cluster randomized trial

**DOI:** 10.1186/s12916-022-02530-x

**Published:** 2022-10-04

**Authors:** Rila Ratovoson, Andres Garchitorena, Daouda Kassie, Jemima A. Ravelonarivo, Voahangy Andrianaranjaka, Seheno Razanatsiorimalala, Avotra Razafimandimby, Fanjasoa Rakotomanana, Laurie Ohlstein, Reziky Mangahasimbola, Sandro A. N. Randrianirisoa, Jocelyn Razafindrakoto, Catherine M. Dentinger, John Williamson, Laurent Kapesa, Patrice Piola, Milijaona Randrianarivelojosia, Julie Thwing, Laura C. Steinhardt, Laurence Baril

**Affiliations:** 1 Epidemiology and Clinical Research Unit, Institut Pasteur de Madagascar, Antananarivo, Madagascar; 2 MIVEGEC, Univ. Montpellier, IRD, CNRS, Montpellier, France; 3 Centre de Coopération International en Recherche Agronomique pour le Développement (CIRAD), Montpellier, France; 4 Malaria Research Unit, Institut Pasteur de Madagascar, Antananarivo, Madagascar; 5 Present address: Humanity & inclusion, Toliara, Madagascar; 6 Present address: Mention Biochimie Fondamentale et Appliquée, Domaine Sciences et Technologie, Faculté des Sciences, Université d’Antananarivo, Antananarivo, Madagascar; 7 U.S. Peace Corps Volunteers, Antananarivo, Madagascar; 8 U.S. President’s Malaria Initiative, USAID, Antananarivo, Madagascar; 9 U.S. President’s Malaria Initiative, Malaria Branch, US Centers for Disease Control and Prevention, Atlanta, GA USA; 10 U.S. President’s Malaria Initiative, US Centers for Disease Control and Prevention, Antananarivo, Madagascar; 11 Epidemiology Unit, Institut Pasteur du Cambodge, Phnom Penh, Cambodia; 12 Faculté des Sciences, Université de Toliara, Toliara, Madagascar

**Keywords:** Malaria detection, Community case management, Rural, Madagascar

## Abstract

**Background:**

Malaria remains a leading cause of morbidity and mortality worldwide, with progress in malaria control stalling in recent years. Proactive community case management (pro-CCM) has been shown to increase access to diagnosis and treatment and reduce malaria burden. However, lack of experimental evidence may hinder the wider adoption of this intervention. We conducted a cluster randomized community intervention trial to assess the efficacy of pro-CCM at decreasing malaria prevalence in rural endemic areas of Madagascar.

**Methods:**

Twenty-two *fokontany* (smallest administrative unit) of the Mananjary district in southeast Madagascar were selected and randomized 1:1 to pro-CCM (intervention) or conventional integrated community case management (iCCM). Residents of all ages in the intervention arm were visited by a community health worker every 2 weeks from March to October 2017 and screened for fever; those with fever were tested by a rapid diagnostic test (RDT) and treated if positive. Malaria prevalence was assessed using RDTs on all consenting study area residents prior to and following the intervention. Hemoglobin was measured among women of reproductive age. Intervention impact was assessed via difference-in-differences analyses using logistic regressions in generalized estimating equations.

**Results:**

A total of 27,087 and 20,475 individuals participated at baseline and endline, respectively. Malaria prevalence decreased from 8.0 to 5.4% in the intervention arm for individuals of all ages and from 6.8 to 5.7% in the control arm. Pro-CCM was associated with a significant reduction in the odds of malaria positivity in children less than 15 years (OR = 0.59; 95% CI [0.38–0.91]), but not in older age groups. There was no impact on anemia among women of reproductive age.

**Conclusion:**

This trial suggests that pro-CCM approaches could help reduce malaria burden in rural endemic areas of low- and middle-income countries, but their impact may be limited to younger age groups with the highest malaria burden.

**Trial registration:**

NCT05223933. Registered on February 4, 2022

**Supplementary Information:**

The online version contains supplementary material available at 10.1186/s12916-022-02530-x.

## Background

Malaria remains a leading cause of morbidity and mortality worldwide. Global control efforts have substantially reduced malaria incidence since 2000, but in 2018, an estimated 228 million cases and 405,000 deaths still occurred, the vast majority in the WHO Africa region [1]. The WHO Global Malaria Programme set a target to reduce malaria incidence and mortality by 90% by 2030 relative to 2015 levels [2]. However, the global malaria burden has remained stable between 2015 and 2017 [3], highlighting the need to intensify prevention and control efforts.

Community health programs have been a cornerstone of malaria control in recent decades. In southeast Asia, the establishment of community health posts in combination with mass administration of antimalarial treatments by community health workers (CHWs) has contributed to malaria elimination efforts [4]. In sub-Saharan Africa (SSA), where transmission is higher, the scale-up of integrated community case management (iCCM) of childhood illnesses has increased access to life-saving interventions, including malaria care, for children under 5 years of age (CU5) [5]. As part of iCCM, CHWs test CU5 with fever using malaria rapid diagnostic tests (RDTs) and treat uncomplicated malaria with artemisinin-based combination therapies (ACTs), often free of charge [6]. The limitation of this community care model is that it excludes individuals older than 5 years and involves only passive detection of febrile cases; many cases remain undetected, increasing the risk of progression to severe disease and death, and contributing to ongoing transmission [5].

To improve prompt malaria care, there is growing interest in proactive community case detection and management of illness by CHWs (proactive community case management, or pro-CCM), whereby households are visited on a regular basis by CHWs for detection and management of health conditions. While the package of interventions offered and populations targeted during these household visits varies depending on the services offered by the organization supporting the CHWs, for the purposes of malaria case management, this refers to the strategy of training and equipping CHWs to visit all the households in the community frequently (weekly to fortnightly), to identify residents of all ages with febrile illness, and to offer malaria rapid testing to those with febrile illness, with treatment for those with positive results. This approach has been piloted in several SSA countries. Initial results of these pilots suggest that pro-CCM could contribute to malaria control [7–9] and, more broadly, reduce child mortality [10]. For instance, a pilot in Senegal involving weekly visits by CHWs to all households in the community for identification and malaria testing of febrile illness with RDTs among residents of all ages and treatment of confirmed malaria resulted in a sixteen-fold reduction of cases after 20 weeks of implementation [7]. The intervention remained effective during a scale-up phase to 16 districts, which revealed rapid increases in diagnosis and treatment rates in the first year of implementation [8]. A similar intervention in Mali with home visits by CHWs at least every 2 weeks, where all febrile CU5 identified were tested for malaria and then treated if RDT positive, more than doubled the rate of early access to antimalarial treatment from 2008 to 2015, while the prevalence of febrile illness among CU5 declined by nearly half in the same period [9]. Even a less resource-intense variation, screening household contacts following a positive RDT in a child detected through iCCM (reactive case detection) was associated with a doubling in the number of cases found in the community in a pilot in Cameroon [11]. Despite these promising results, there is no experimental evidence of the effects of pro-CCM on malaria prevalence [5, 10], which may hinder its wider adoption for malaria control in endemic areas.

Madagascar could benefit from pro-CCM-like interventions to improve malaria control. Malaria remains a leading cause of mortality on the island [12]. After an initial decline in malaria incidence in the 2000s, this trend reversed, with increases in malaria cases in many areas since 2009 [13]. Madagascar’s health system provides iCCM for CU5 via a network of over 30,000 CHWs [14–16] and free malaria diagnosis and treatment across all levels of the health system. However, health-seeking behaviors and access to malaria diagnosis and treatment remain extremely low [17]. Among children with reported fever in a 2016 national malaria survey, only 46.2% sought care, 15.5% had an RDT done, and 10.1% received an antimalarial [18]. Thus, additional strategies are necessary to increase access to malaria diagnosis and treatment, reduce the disease burden and limit transmission.

To compare the effect of pro-CCM to conventional (passive) CCM on malaria prevalence in rural Madagascar, we conducted a cluster randomized community intervention trial in a southeastern district of the country: Mananjary district. The intervention was based on the proactive model implemented in Senegal [7] and involved systematic visits to all households in a CHW’s catchment area every 2 weeks to identify all residents with fever, test them with an RDT, and treat those with positive results.

## Results

### Characteristics of study participants and individual-level malaria risk factors

**Fig. 1:**
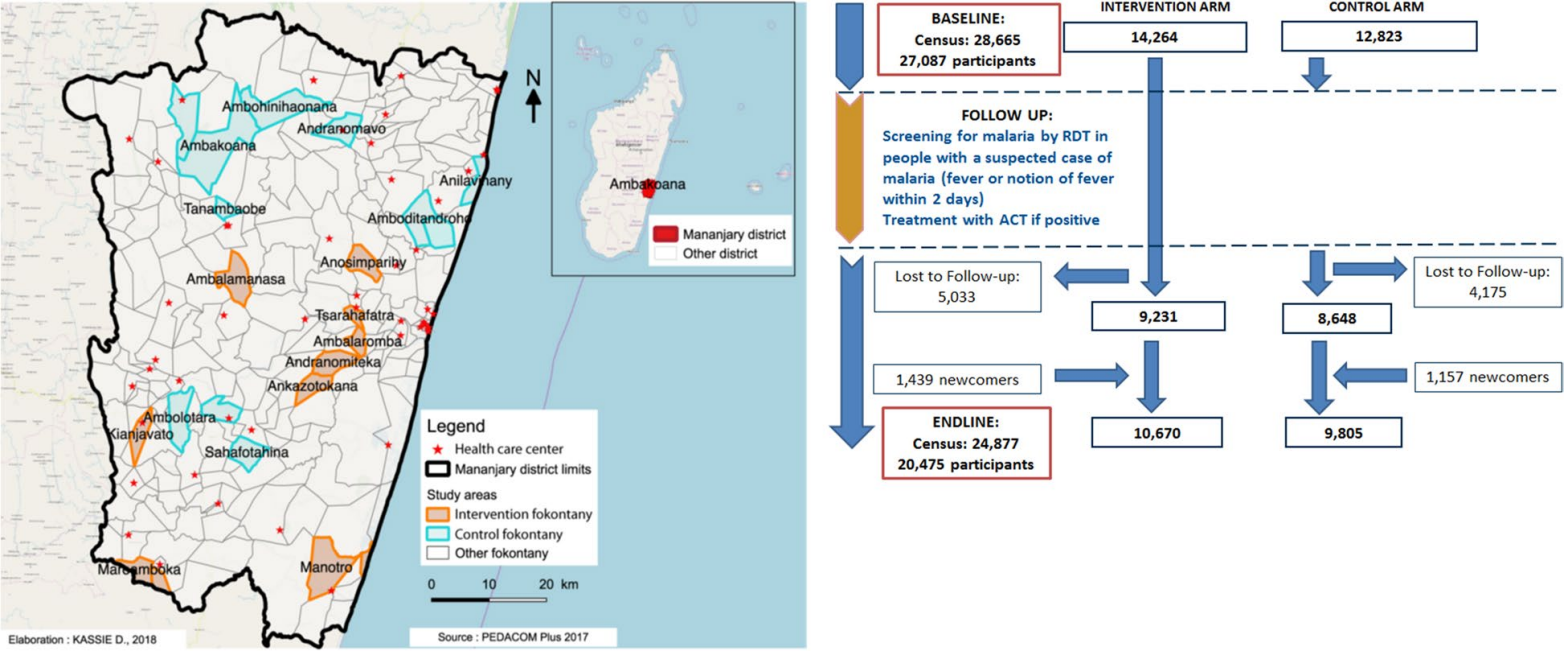
Study design of the pro-CCM cluster randomized trial in the Mananjary district. Left: map of the Mananjary district and the fokontany randomized to the intervention and control arms. Right: recruitment of study participants in each arm at baseline, follow-up, and endline

In total, 6406 households comprised of 28,665 individuals were registered in the 22 *fokontany* during the household census (Fig. 1). Of these individuals, 27,087 (94.5%) agreed to participate during the baseline survey and be screened for malaria (14,264 in the intervention and 12,823 in the control arm). During the endline survey, 4995 households, containing 24,877 residents, were contacted by the interviewers (Fig. 1). The remaining baseline households were not present at the time of the second census. Of the individuals visited, 20,475 (82.3%) agreed to participate in the endline survey. Overall, 17,879 of 27,087 (60.0%) residents from the baseline survey (9231 from the intervention arm and 8648 from the control arm) participated in the endline survey. In addition, 2596 new residents (1439 in the intervention arm and 1157 in the control arm) participated. These new residents were either new arrivals in a household who participated during baseline (in both arms) or new households who were included during the study implementation in the intervention arm (Fig. 1). Those who were in the baseline but not the endline census had either moved or were absent, died, or refused to participate (Additional file 1: Fig. S1).

The mean age of the study population at baseline was 20.3 years (sd = 17.6); 49.1% were under the age of 15 years, and 53.8% were female (Table 1). Among participants > 18 years, 7162 (59.0%) had completed primary school or less. Participation was different in baseline and endline surveys by intervention status, but the socio-demographic characteristics of the participants were similar in the two arms, both at baseline and endline (Table 1). Malaria prevalence at baseline was 7.4% in the study clusters (95% CI [7.1–7.8%]) with a higher prevalence in the intervention arm (8.0%, 95% CI [7.6–8.4%]) than in the control arm (6.8%, 95% CI [6.4–7.3%]). *Fokontany*-level prevalence ranged from 1.1 to 19.5% in the intervention arm and from 3.0 to 11.2% in the control arm (Additional file 2: Table S1). By the end of the study, malaria prevalence had decreased to 5.5% (95% CI [5.2–5.8%]) in the study clusters, with a lower prevalence in the intervention arm (5.4%, 95% CI [4.9–5.8%]) compared to the control arm (5.7%, 95% CI [5.2–5.8%]).

**Table 1:** Characteristics of the population participating in the baseline and endline surveys, Mananjary, Madagascar

Characteristics	Baseline						Endline					
	Intervention arm *N* = 14,264		Control arm *N* = 12,823		Total *N* = 27,087		Intervention arm *N* = 10,670		Control arm *N* = 9805		Total *N* = 20,475	
	*n*	*%*	*n*	*%*	*n*	*%*	*n*	*%*	*n*	*%*	*n*	*%*
Age group (years)												
0–4	2638	*18.5*	2644	*20.6*	5282	*19.5*	1976	*18.5*	1987	*20.3*	3963	*19.4*
5–14	4220	*29.6*	3792	*29.6*	8012	*29.6*	3247	*30.4*	2936	*29.9*	6183	*30.2*
15–49	5934	*41.6*	5376	*41.9*	11,310	*41.8*	4264	*40.0*	4034	*41.1*	8298	*40.5*
>50	1472	*10.3*	1011	*7.9*	2483	*9.2*	1183	*11.1*	848	*8.6*	2031	*9.9*
Sex												
Female	7823	*54.8*	6744	*52.6*	14,567	*53.8*	5885	*55.2*	5191	*52.9*	11,076	*54.1*
Male	6441	*45.2*	6079	*47.4*	12,520	*46.2*	4785	*44.8*	4614	*47.1*	9399	*45.9*
Educational level for participants ≥ 18 years												
No school	1605	*24.7*	1703	*30.2*	*3308*	*27.2*	1141	*23.7*	1294	*30.1*	2435	*26.7*
Primary	3896	*60.0*	3266	*57.9*	*7162*	*59.0*	2953	*61.4*	2529	*58.9*	5482	*60.2*
Secondary	892	*13.7*	623	*11.0*	*1515*	*12.5*	657	*13.7*	423	*9.9*	1080	*11.9*
University	104	*1.6*	52	*0.9*	*156*	*1.3*	60	*1.2*	47	*1.1*	107	*1.2*
Sometimes sleeps outside the house												
Yes	173	*1.2*	160	*1.2*	333	*1.2*	88	*0.8*	90	*0.9*	178	*0.9*
Sleeps under an LLIN every night												
Yes	12,689	*89.0*	11,599	*90.5*	24,288	*89.7*	9973	*93.5*	9027	*92.1*	19,000	*92.8*
Malaria RDT positivity												
Yes	1141	*8.0*	875	*6.8*	2016	*7.4*	574	*5.4*	560	*5.7*	1134	*5.5*

In the baseline survey, children 5 to 14 years of age had over twice the odds of being RDT positive than CU5 (OR = 2.48; 95% CI [2.17–2.83]). Males had a higher odds of RDT positivity than females (OR = 1.26 95% CI [1.15–1.39]), and subjects who had never attended school had increased odds compared to those with at least some schooling (Table 2). Protective behaviors such as sleeping under an LLIN every night were associated with a lower odds of malaria positivity (Table 2). After accounting for individual-level fixed effects and random effects at the *fokontany* level, there were no statistical differences at baseline in RDT positivity between individuals living in *fokontany* randomized to the intervention or control arm (OR = 0.89; 95% CI [0.46–1.70]). Individual risk factors for malaria for the endline population (captured in questionnaires at baseline or at the time of inclusion during pro-CCM implementation) had similar associations to malaria positivity as those of the baseline population, both in terms of statistical significance and effect size (Table 2).

**Table 2:** Individual and household-level predictors of RDT positivity in the baseline and endline surveys (multivariate results, generalized linear mixed model)^a^

Characteristics	Baseline	Endline
	OR (95% CI)	OR (95% CI)
Intercept	0.11 (0.07–0.17)***	0.09 (0.05–0.15)***
**Intervention arm**		
Intervention arm (vs control)	0.89 (0.46–1.70)	0.71 (0.36–1.43)
**Socio-demographic characteristics**		
Sex (male vs female)	1.26 (1.15–1.39)***	1.32 (1.16–1.49)***
Age group, years (ref. 0–4)		
5–14	2.48 (2.17–2.83)***	2.35 (1.96–2.81)***
15–49	0.77 (0.67–0.89)***	1.02 (0.84–1.23)
>50	0.35 (0.26–0.47)***	0.39 (0.27–0.58)***
Highest educational level among participants ≥ 18 years (ref. no school)		
Primary school	0.72 (0.63–0.81)***	0.78 (0.66–0.92)**
Secondary school	0.66 (0.56–0.79)***	0.57 (0.45–0.71)***
University	0.23 (0.13–0.39)***	0.56 (0.32–0.98)*
**Protective behaviors**		
Sleeps under an LLIN every night	0.79 (0.68–0.92)**	0.42 (0.35–0.51)***

*p-value < 0.05; **p-value < 0.01; ***p-value < 0.001

^a^Separate regression models were run for baseline and endline data

### Pro-CCM implementation follow-up and impact on malaria prevalence

**Fig. 2:**
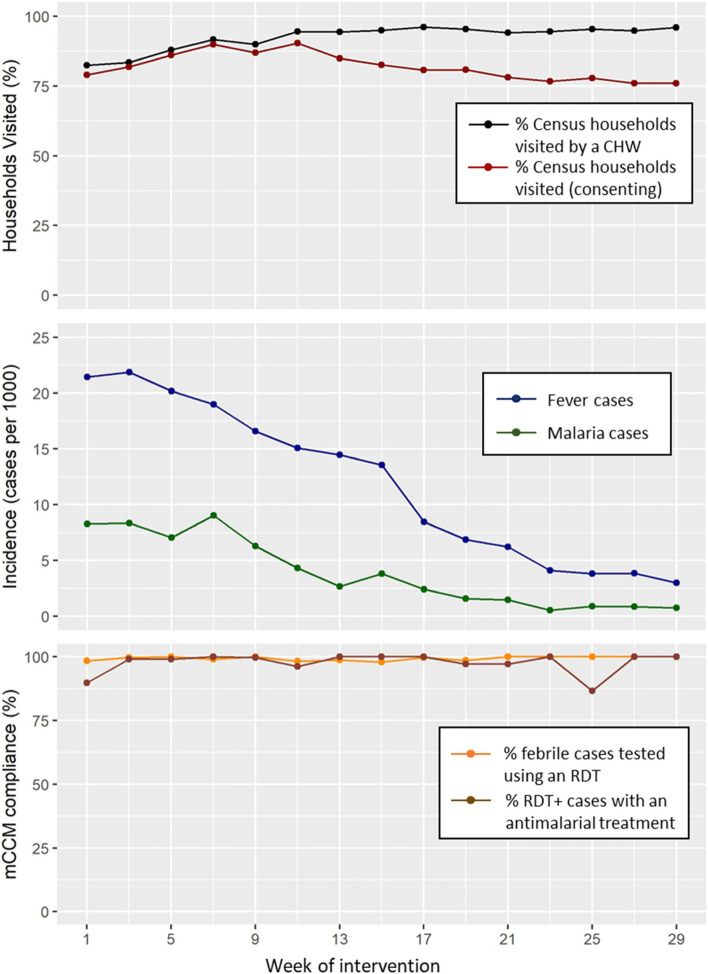
Follow-up of pro-CCM implementation, from March to October 2017. Graphs show the evolution of average values for fokontany in the intervention arm, estimated at each visit (every 2 weeks), with colors representing different indicators

During the pro-CCM intervention period, from March to October 2017, CHWs conducted 15 biweekly visits to households in their *fokontany* catchment area. During each of the 15 visits, 80–100% of households were visited on average, and 60–95% of households agreed to participate (Table 3). The percentage of households visited increased over time; by the middle of the study period, over 95% of households were visited. However, the percentage of households consenting to the CHW visit decreased from the middle of the study period towards the end, reaching a low of 75% (Fig. 2). Fever and malaria incidence varied by *fokontany* during the intervention period but steadily decreased, from an average of >20 to <5 cases of fever per 1000 people per visit (every 2 weeks) and from about 9 to <1 cases of malaria per 1000 people (Fig. 2). Rates of fever and malaria also decreased at the health center level in Mananjary district during this period, but at a slower rate than observed at the community level in the intervention arm (Additional file 1: Fig. S2). CHW compliance with pro-CCM screening and treatment protocols were close to 100% throughout the study period (Fig. 2) for nearly all *fokontany* (Table 3). The pro-CCM intervention substantially increased the rates of detection and treatment of malaria cases, as compared with passive detection via iCCM in either the control or intervention arm (Additional file 1: Fig. S3). The combination of iCCM and pro-CCM in the intervention arm led to a 4-fold increase in the number of RDTs done and a 3-fold increase in the number of ACTs delivered as compared with iCCM alone in the 2 years prior (Additional file 1: Fig. S3).

**Table 3:** Fokontany-level process indicators^a^ of pro-CCM intervention activities, Mananjary District, Madagascar, March–October 2017

Fokontany in the intervention arm	% Census households visited by a CHW every 2 weeks (out of all households listed)	% Census households consenting to screening (out of all households listed)	Incidence of fever per 1000 pop. (out of all individuals listed)	Incidence of malaria per 1000 pop. (out of all individuals listed)	% febrile cases tested using a malaria RDT	% RDT+ cases with an antimalarial treatment
Kianjavato	86.3	74.8	3.8	1.2	100	100
Ambinany Namorona	96.4	94.2	16.6	5.2	98.8	100
Manotro	96.3	94.9	16.4	5.4	98.4	97.8
Anosiparihy	98.9	82.4	15.3	2.8	100	94.8
Ambalamanasa	84.2	59.2	4.5	0.7	100	87.5
Tanambao Sud	81.8	76.5	6.2	1.3	95.4	100
Maroamboka	80.0	65.5	7.4	2.5	99.6	93.9
Ankazotokana	97.2	89.6	23.3	11.8	99.8	99.8
Tsarahafatra	97.1	87.7	8.4	1.2	100	100
Ambalaromba	100	91.4	14.3	3.3	100	100
Andranomiteka	97.6	84.2	14.7	6.8	99.7	98.6

^a^All indicators were estimated as the average per visit (every 2 weeks) over the 8-month intervention period

**Fig. 3:**
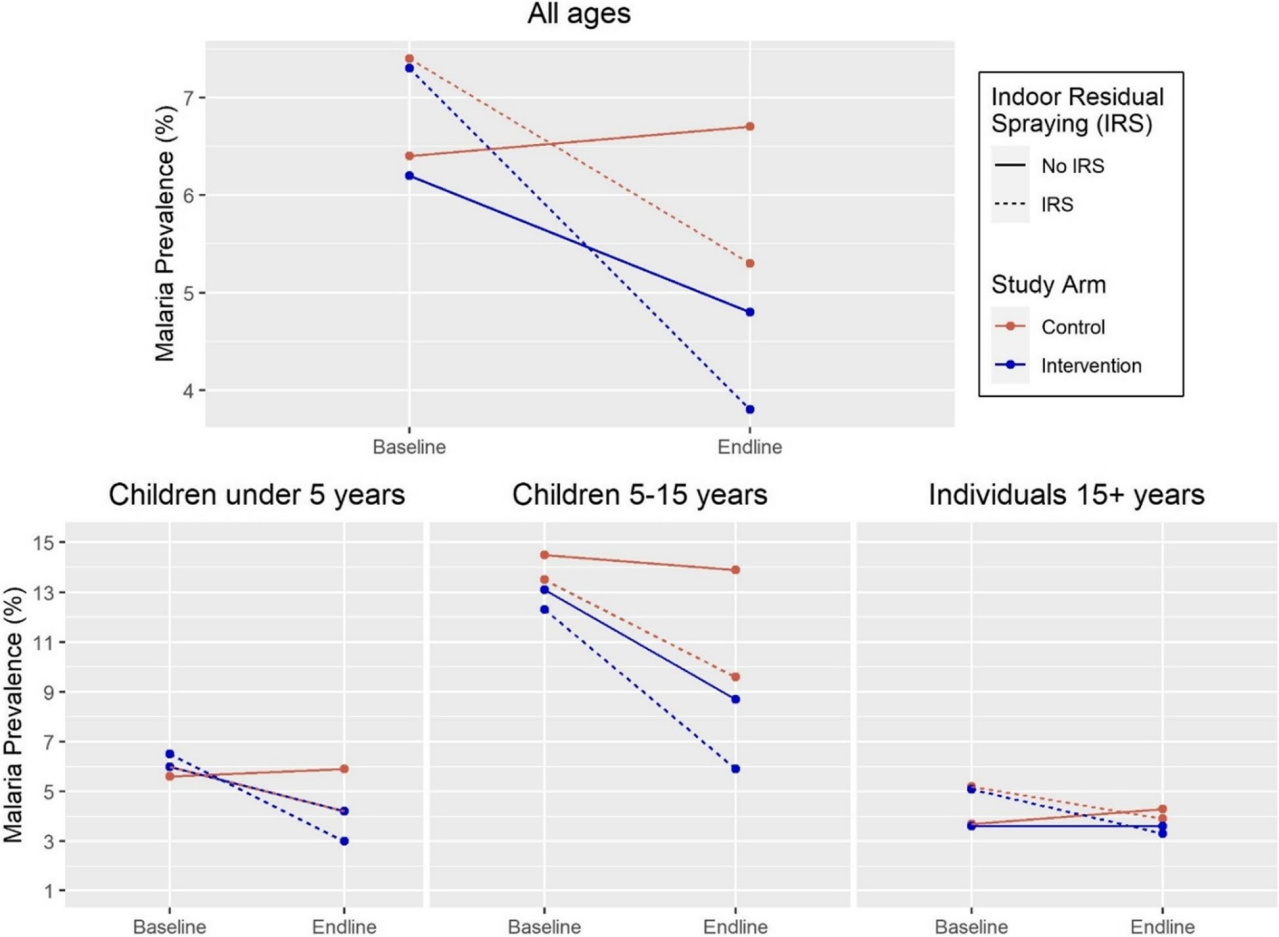
Impact of pro-CCM on malaria prevalence. Graphs show the predicted change in malaria prevalence over the study period in the intervention and control arms, for the whole population and particular age groups. In-sample predictions were obtained from multivariate models by age group described in Table 4. Colors represent study arms, and dashed lines represent changes in fokontany receiving IRS

Intention-to-treat difference-in-differences analyses revealed that the intervention was associated with a significant reduction in malaria prevalence only for children 5 to 14 years (OR = 0.66; 95% CI [0.47–0.91]), the age group with the highest baseline prevalence (Table 4). A similar effect was observed among CU5, but this effect was not significant (OR = 0.65; 95% CI [0.28–1.54]), while for individuals of all ages the reduction was less pronounced (OR = 0.72; 95% CI [0.49–1.06]) and only borderline significant (*p* < 0.1). Modeling the effect for all children under 15 years revealed the largest significant reduction in malaria prevalence associated with the intervention (OR = 0.59; 95% CI [0.38–0.91]). For individuals 15 years of age and older, the reduction was substantially smaller and not significant (OR = 0.87; 95% CI [0.54–1.39]). IRS implementation was associated with independent significant reductions in malaria prevalence in most age groups, with ORs ranging from 0.63 to 0.71 depending on the age group considered, so that *fokontany* where both interventions were implemented experienced the largest reductions. In-sample predictions of pro-CCM intervention and IRS impact are displayed in Fig. 3.

**Table 4:** Impact of pro-CCM and IRS on malaria prevalence, intention-to-treat analyses (multivariate results, generalized estimating equations)

Variable	Individuals all ages^a^	Children less than 15 years^b^	Children under 5 years	Children 5 to 14 years	Individuals 15+ years
	Adjusted OR (95% CI)	Adjusted OR (95% CI)	Adjusted OR (95% CI)	Adjusted OR (95% CI)	Adjusted OR (95% CI)
Intercept	0.05 (0.03–0.08) ***	0.14 (0.05–0.38)**	0.06 (0.03–0.1) ***	0.14 (0.09–0.22) ***	0.04 (0.02–0.06) ***
**Differences at baseline**					
Between arms (intervention vs. control)	0.98 (0.52–1.82)	0.29 (0.05–1.65)	1.08 (0.57–2.06)	0.89 (0.49–1.99)	0.97 (0.54–1.73)
Between IRS status (receiving vs. not)	1.17 (0.6–2.29)	0.39 (0.07–2.13)	1.08 (0.46–2.57)	0.93 (0.48–1.77)	1.42 (0.83–2.45)
**Differences over time**					
Endline vs. baseline	1.04 (0.76–1.42)	1 (0.85–1.19)	1.06 (0.59–1.91)	0.96 (0.75–1.22)	1.16 (0.77–1.73)
**Impact of interventions**					
Impact of pro-CCM over time (DiD)	0.72 (0.49–1.06)	0.59 (0.38–0.91)*	0.65 (0.28–1.54)	0.66 (0.47–0.91) *	0.87 (0.54–1.39)
Impact of IRS over time (DiD)	0.66 (0.44–0.98) *	0.65 (0.47–0.92)*	0.65 (0.27–1.54)	0.71 (0.52–0.97) *	0.63 (0.4–1.01)

*p-value < 0.05; **p-value < 0.01; ***p-value < 0.001

^a^Model adjusted for age group; children 0–4 years (ref); children 5–14 years OR 2.49 (95% CI 1.93–3.21)***; individuals 15+ years OR 0.76 (95% CI 0.54–1.06)

^b^Model adjusted for age group; children 0–4 years (ref); children 5–14 years OR 2.33 (95% CI 1.98–2.73)***

Per-protocol analyses, which included only the subset of 17,879 individuals who participated in both baseline and endline surveys, had results consistent with intention-to-treat analyses in terms of the magnitude on pro-CCM and IRS impact, but they were not statistically significant (Additional file 2: Table S2).

The intervention had no impact of the prevalence of anemia among women of reproductive age (Additional file 3).

## Discussion

The recent increase in malaria morbidity and mortality in multiple SSA countries highlights the urgent need for additional strategies to improve malaria control in high-transmission settings [19]. Following observational studies from Senegal and Mali of pro-CCM for malaria, there are indications that this intervention can be effective at increasing rates of malaria diagnosis and treatment and at reducing malaria burdens [7, 9]. Using a cluster randomized community intervention design in a rural endemic area of Madagascar, here, we provide the first experimental evidence that pro-CCM can help reduce malaria prevalence in a moderate transmission setting, but its effect may be limited to certain high-risk groups. In less than 1 year of implementation, the intervention was associated with a significant reduction in the odds of malaria positivity for children under 15 years, even after adjusting for IRS implementation, but not for individuals 15 years or older. Malaria prevalence decreased from 8.0 to 5.4% in the intervention arm for individuals of all ages, but only from 6.8 to 5.7% in the control arm. The largest effect was observed when combining IRS with proactive community case management.

The results from this trial fill a critical gap in the evidence available to inform decisions regarding the scale-up of pro-CCM interventions. Indeed, measuring the effects of home-based care delivery models with robust study designs that allow for causal attribution is one of the key research priorities identified by the WHO in its most recent guideline on CHW programs [20]. In 2019, a systematic review of proactive case detection interventions showed that while they are likely to reduce child mortality, their impact on the prevalence of childhood illnesses is still inconclusive [10]. For malaria control specifically, another systematic review suggested that community-based models are better at improving access to and timeliness of treatment, but there is substantial heterogeneity in the implementation models, study designs, and results [5]. Of 28 studies included in the review, only 4 were cluster randomized trials involving home-based care (by mothers, not CHWs), and just one from Burkina Faso assessed the effect on malaria and fever prevalence, finding no significant impacts on either [5]. Currently, experimental studies are underway to rigorously assess the impact of pro-CCM interventions, such as a pragmatic effectiveness-implementation evaluation (including stepped-wedged cluster randomization) in Togo [6], and a cluster randomized trial in rural Mali [21], both focused on child survival. In this Madagascar trial, pro-CCM helped reduce malaria prevalence, but the effect was only statistically significant among children less than 15 years, suggesting that its impact for older age groups with lower levels of infection may be limited. The lack of effect in adults could be due to the lower baseline malaria prevalence in this group, as well as biological and behavioral factors. For instance, higher levels of immunity in adults likely result in fewer symptomatic infections in this population. Additionally, adults may be more likely to be working their fields despite having a fever and potentially being infected with malaria, and thus, infections would not be detected during household screening visits.

The results presented here are consistent with previous evidence from Senegal and Mali, albeit with some differences. The Senegal study found a much larger reduction (16-fold) in the point prevalence of symptomatic confirmed malaria infection in the intervention arm compared to the control arm after 20 weeks of implementation but did not measure parasite prevalence in the general population. Moreover, household visits with fever screening in the Senegal program were conducted weekly, and case management was offered to all ages in both arms when residents sought care from CHWs. In the Mali model, analyzed by interrupted time series, case management was also offered to all ages, while the timing of visits was more flexible (at least every 2 weeks) [9]. After 7 years, the proportion of children under 5 years reporting fever in the previous 2 weeks decreased from 40 to 23%, though malaria incidence and prevalence were not reported. Both in Mali and in Senegal, offering pro-CCM dramatically increased the number of malaria cases diagnosed and treated by CHWs, suggesting that most cases had been going untreated, contributing to ongoing transmission. This is in line with our findings, where the incidence of fever and malaria as estimated from pro-CCM data was about twice the incidence estimated from district health facilities (Additional file 1: Fig. S2) and the combination of iCCM and pro-CCM resulted in a 4-fold increase in the number of RDTs done and a 3-fold increase in the number of ACTs delivered, as compared with iCCM alone in the 2 years prior (Additional file 1: Fig. S3).

The scale-up of a sustainable pro-CCM approach in areas of moderate malaria transmission will require careful planning, dedicated resources, and political buy-in. A recent evaluation showed that most intervention pilots are not scaled-up, even after demonstrating conclusive improvements in malaria control [22]. Yet, the experience of Senegal, which scaled-up pro-CCM to 24 districts between 2014 and 2017, and has since been expanded even further, suggests that implementation at scale is both possible and sustainable. In the first year of implementation, rates of diagnosis and treatment by CHWs tripled, including a doubling in the rates of care seeking at community sites, despite a modest increase of 7% in the number of CHWs [8]. In Madagascar, several important actions will be necessary, including community sensitization, CHW training and supervision, and financial compensation, as CHWs risk overload due to their involvement in several health programs and the lack of formal salary support. A key component of the pro-CCM intervention was to distribute enough malaria supplies to CHWs to prevent stock-outs during the implementation period. Given challenges in supply chain management for malaria commodities across Madagascar [23], solutions need to be devised to ensure reliable stocks of RDTs and ACTs for health centers and CHWs. Additional challenges include persistent geographic barriers in access to care (e.g., poor road networks, remote populations, and limited health facilities) and security issues in many rural areas of Madagascar. To ensure favorable study implementation conditions, cluster selection in this study excluded some areas that would have been very challenging to access. In a pro-CCM scale-up, there could be some reduction in effectiveness, particularly in these remote areas. Responding to all these challenges will require significant additional investments from the Ministry of Public Health and its partners.

An important finding of this study was the higher prevalence of malaria observed in children 5–14 years, who had more than twice the prevalence of children under 5 years. While iCCM for children under 5 years has been in place in Madagascar for several years, there is currently no community-based strategy targeting children older than 5 years. This can limit this group’s access to malaria diagnostics, treatment, prevention, and sensitization. For instance, children 5–14 years had the lowest bed net use in our surveys of any age group (Additional file 2: Table S4). As a result of this access gap, the burden of malaria may have shifted to older children, making this age group an important reservoir of malaria infections. This has been observed in other settings [24]. Our results suggest that pro-CCM interventions can be an effective way to reduce malaria burdens for this age group, where it had the largest impact. However, given the resources necessary for the scale-up of pro-CCM, other less resource-intensive alternatives could be envisioned. For instance, school-based programs or an expansion of the target age for passive malaria case management in the community might also be efficient ways to diagnose and treat malaria in older children and adolescents. Indeed, expanding the age of malaria community case management to individuals older than 5 years is part of the malaria operational plan in Madagascar and is currently being assessed in a separate cluster randomized trial [25].

This study had several limitations. First, the project was conducted in remote rural areas of the country where accessibility (among other logistical issues) was particularly difficult during the rainy season, and thus required additional study staff and CHWs to ensure appropriate implementation. This could explain the initially low percent of census households visited, which improved over the first 3 months of intervention. Second, while study staff collected data from CHWs in the intervention arm during intervention implementation, no information was collected from standard iCCM forms from CHWs in either arm during study implementation. This information was collected after the study was completed from available sources, which affected data completeness (13 fokontany out of 22, with 75% of months available between March 2015 and October 2017). Still, the high rates of CHW adherence to the intervention were consistent with higher rates of detection during pro-CCM as compared to passive detection during iCCM and at health centers, which suggests that the intervention contributed to a substantial increase in access to malaria diagnosis and treatment overall. Third, a large decrease in the number of participants from the baseline to the endline survey was observed for both arms. Many participants were unreachable because they were working on the clove harvest (the main source of income in this area), which coincided with the endline survey, and some individuals included in the baseline refused to participate in the endline survey due to research fatigue. In addition, CHWs may have lacked the motivation to visit households two or three times to find the study participants or to encourage their participation, and this could have an implication in any scale-up of this intervention. However, both baseline and endline populations had similar characteristics, and effects obtained from intention-to-treat or per-protocol analyses were consistent, so it is unlikely that this decrease in sample size biased the results of the trial. Finally, most RDT-positive individuals surveyed at baseline in both arms were treated with an ACT, in compliance with ethical standards. This may have forced some equivalence between the two arms at the beginning of the intervention and could explain the lower than expected effect of pro-CCM in our trial.

## Conclusion

The results from this study highlight that proactive malaria community case management strategies can contribute to improving malaria control in moderate-transmission settings, but the effects observed here were smaller than anticipated or reported elsewhere. The largest effect was on malaria prevalence among children 5–14 years, the age group with the highest parasite prevalence in this setting and not typically targeted in conventional iCCM strategies. The external validity of these results will need to be confirmed with similar studies in other countries. In the meantime, a scale-up to other districts in Madagascar will require clear criteria to identify areas that could benefit the most given the additional resources required. Alternatively, less resource-intensive strategies such as expanding the age of malaria community case management to older children and adolescents could be considered.

## Methods

### Study area

The study was conducted in 22 rural *fokontany* (smallest administrative unit comprising one or several villages) in the Mananjary district of the Vatovavy Fitovinany region, in southeast Madagascar (Additional file 1: Fig. S4). The total population of the Mananjary district was estimated at 334,331 inhabitants in 2015, distributed in 150 *fokontany*. The mean population density has doubled in the last 20 years, from 37 inhabitants/km^2^ in 1995 to 74 inhabitants/km^2^ in 2015 [26]. Southeast Madagascar has among the highest malaria burdens in the country, and the Mananjary district had the highest number of reported malaria outbreaks in the country from 2012 to 2015 [23]. After the 2015 long-lasting insecticidal net (LLIN) campaign, 87% of the population in this region reported sleeping under an LLIN [18], and indoor residual spraying (IRS) campaigns were done in 2017 to reduce malaria transmission.

As part of the Madagascar national policy for community health, the routine tasks of CHWs in Mananjary include the diagnosis and treatment of malaria, diarrhea, and acute respiratory infections in CU5 following international iCCM protocols. In addition to the high burden of malaria in the district, Mananjary was selected for the pro-CCM study because of the presence of Peace Corps Volunteers (PCV) assigned to the district who could support study implementation.

### Inclusion criteria, field preparation, and randomization

The criteria for *fokontany* selection included a location in a rural area of Mananjary and the agreement of the village chief for the *fokontany* to participate. *Fokontany* with a population of less than 1000 inhabitants and those located in an urban area or in an area where access by study teams was unsafe were excluded. Following randomization (see below), individuals from all households in the selected *fokontany* who agreed to participate in the study were included. Only households providing written informed consent were included in the study, and only individuals providing verbal consent for interviews and testing were included. Written consent was obtained from parents or guardians for children under 18 years old. Individuals in the intervention arm who arrived after the study started were invited to participate in the pro-CCM intervention.

Once approval for the study was obtained from the Malagasy National Ethics Committee (CERBM n°103-MSANP/CE on September 12, 2016), courtesy visits were made to all administrative and health officials in Mananjary.

The study was designed to detect a 50% decrease in malaria prevalence at endline (10% to 5%) in the intervention arm relative to the control arm. Considering an *α* of 0.05, a power of 80% for a two-tailed test, and an intracluster correlation coefficient (ICC) of 0.02, we included 22 clusters (11 intervention and 11 control *fokontany*). The sample size was calculated using the “Sample Size Estimation Functions for Cluster Randomized Trials (CRTSize)” package [27] in R version 3.5.2 [28], assuming that at least 1000 inhabitants would be included in each cluster (one of the inclusion criteria for *fokontany*).

Of 150 *fokontany* listed in the district, 124 fulfilled eligibility criteria; from these, 30 were selected at random (15 per arm, with four backup *fokontany* per arm) for a target of 11 *fokontany* per arm, using the *sample* function in R software version 3.5.2 [28]. Contiguous *fokontany* assigned to different arms were removed to prevent potential contamination. A second draw was performed to replace the *fokontany* removed, again with contiguous *fokontany* belonging to different arms removed to obtain the final set of 15 non-contiguous *fokontany* per arm. Field visits were then made to the 30 *fokontany* to identify the 11 *fokontany* per arm (Fig. 1) to retain for the study. During this step, population data were verified on site using *fokontany* records to confirm that the current population was indeed over 1000 people; the level of acceptance by the village chief and CHWs to be part of the study was checked; and *fokontany* level of safety, accessibility by study teams, and phone network availability were also assessed. *Fokontany* that were landlocked during the rainy season or that required more than a day’s travel on foot were deemed poorly accessible. *Fokontany* were ranked according to the population size, acceptance, safety, accessibility, and phone network and those that best met the criteria were retained.

The *fokontany* in the study area were in the catchment of 10 health care centers (HCCs) (Fig. 1).

### Study design

The study was a two-arm cluster randomized community intervention trial, with 11 *fokontany* in the intervention arm implementing malaria pro-CCM and 11 in the control arm. The main objective was to assess the efficacy of pro-CCM on reducing malaria prevalence. In both arms, CHWs provided passive iCCM among CU5 per usual standard of care, including diagnosis with RDT for febrile illness, treatment with artesunate-amodiaquine according to RDT results, along with diagnosis and management of acute respiratory infections and diarrhea, and referral to a higher level of care if indicated. Oral rehydration salts and antibiotics were distributed to CHWs in the intervention arm to reinforce the iCCM activities already implemented.

In addition to routine iCCM activities, CHWs in the intervention arm also conducted door-to-door fever screening for all inhabitants of all consenting households in their catchment area every fortnight. All individuals with temperature ≥ 37.5°C or a history of self-reported fever in the previous 2 weeks were tested with an RDT; positive individuals who were not pregnant and did not have signs of severe disease were treated with artesunate-amodiaquine by the CHW according to treatment guidelines. Individuals identified as requiring a referral during pro-CCM visits were assisted with transfer to the HCC, with transportation handled by the project staff. CHWs followed up individuals referred to HCC after approximately 2 days. In between biweekly household visits, case management was provided to CU5 via conventional iCCM; those > 5 years sought care at the HCC or waited until the next household visit. During the pro-CCM household visits, verbal informed consent was obtained before fever screening; parents or guardians were asked to give consent for screening of children under 12 years. Intervention follow-up was initially planned in the study protocol to last for 12 months, but due to delays in intervention kick-off and duration of surveys, in order to be able to compare prevalence during the same transmission season in baseline and endline surveys, the intervention follow-up was reduced to 8 months.

IRS was carried out in four *fokontany* in the intervention arm and six in the control arm from July 24 to August 21, 2017 (during the pro-CCM intervention follow-up). IRS was implemented by the PMI-VectorLink project, independent of the study, so balance in IRS implementation between the study arms was not considered during the study design phase.

### Pro-CCM implementation

Health workers at HCCs and CHWs in the 22 fokontany included in the study were trained in the project objectives, methodology, data collection, distribution, and supervision. CHWs in both arms were trained to conduct baseline and endline surveys and received refresher training on case management for uncomplicated malaria among CU5. CHWs in the intervention arm received additional training on pro-CCM for individuals of all ages. After training, each intervention CHW was equipped with a solar charger, a cell phone, artemisinin-based combination therapy (ACTs), an axillary electronic thermometer, antipyretic medications, RDTs, sharps containers, and gloves. Procurement for conventional iCCM was strengthened in the intervention arm, with oral rehydration salts and cotrimoxazole being distributed to CHWs. Study tracking forms were given to intervention CHWs for data collection during the household visits; CHWs in both arms used their usual patient registers when caring for CU5 during conventional iCCM visits.

During pro-CCM implementation, from March to October 2017, CHWs and HCCs in both arms were provided with malaria RDTs, antipyretics, and ACTs each time they ran out of stocks to ensure adequate supplies. A United States Peace Corps Volunteer assisted with study supervision, especially during baseline and endline data collection. Six clinical research assistants (CRAs) hired for the study supervised CHWs in both arms. Particular attention was given to supervision in *fokontany* with more logistical constraints such as villages with difficult access during the rainy season. In addition, the limited availability of some CHWs due to pregnancy or other work commitments led to the recruitment of four additional CHWs (from 22 to 26 CHWs). The six CRAs were responsible for ensuring an uninterrupted supply of RDTs and ACTs. All CHWs in the intervention arm were paid an incentive of 70,000 MGA (~22.5 USD) per month for the additional pro-CCM work.

### Data collection and management

#### Survey data collection at baseline and endline

The baseline survey was conducted from December 2016 to February 2017 in all 22 *fokontany*. The survey was conducted by a team of 22 field data collectors and 6 supervisors. For each fokontany, a population census and GPS coordinates of all buildings in the *fokontany* were recorded into an electronic database. For each household, a questionnaire was administered to the head of the household to collect socio-demographic household composition, education level and employment status of the head of the household, and asset ownership information. For each consenting household member, a fever screening, which included measuring temperature with an electronic thermometer and asking about fever within the previous 2 days, was conducted. For household members with suspected malaria (i.e., current or recent fever), a symptom questionnaire was completed (for children from 2 months to 18 years, guardians assisted with data collection). All consenting *fokontany* residents were tested by RDT regardless of symptoms, and nearly all (97.2%) of those testing positive received an ACT treatment, with the exception of pregnant women with positive RDTs who were referred to the nearest HCC and 49 persons whose reason for refusal was not documented. Residents with fever received antipyretics. Women aged 15 to 49 years were screened by hemoglobinometer (HemoCue Hb 201+) and received an iron-folic acid treatment if severe anemia was detected (less than 80g/L in non-pregnant women and less than 70g/L in pregnant women). Anemia classification thresholds depended on pregnancy status, which was obtained by self-report during the interview. Household residents who were absent during the first survey visit were visited within 3 days. Households and individuals in the intervention arm who decided to participate after the intervention started were administered a baseline survey at the time of study inclusion. No new households were included during the endline in either arm; only individuals who were new residents of an already participating household in either arm were added during the endline survey.

After 30 weeks of intervention (15 biweekly visits), an endline survey among all baseline households was conducted from November 2017 to January 2018. Survey methods were identical to the baseline survey; however, the information collected was limited to recent symptoms of malaria as household-level socio-demographic data had been collected at baseline or at the time of study inclusion for new households in the intervention arm.

A period of censure of 1 month was established between the baseline cross-sectional survey and the start of the pro-CCM intervention and between the end of the pro-CCM intervention and the endline cross-sectional survey.

#### Health data collection during the intervention

In the intervention arm, data was collected by CHWs on all visited consenting households every 2 weeks, which was included in a household file. CHWs asked about fever or recent fever to the person who knew all members of the household best (usually the wife); when fever was reported, CHWs recorded individual fever and RDT screening data on paper forms, and these were added to the household file. Conventional iCCM visit data collection was not included for either arm as part of this study. This information was obtained after study completion from the USAID Mikolo project, which had provided support to CHWs for iCCM activities (including data collection) in fokontany further than 5 km from health facilities in our study area during a period that comprised the pro-CCM intervention. We requested available data for the intervention period (March to October 2017) and 2 years prior (March 2015 to February 2017), which included 6 fokontany in the intervention arm and 7 fokontany in the control arm.

#### Data management

Baseline and endline survey data were collected on electronic tablets with AlcatelPixi 4 software. Biweekly visit data from the intervention arm were collected on paper forms by CHWs and entered electronically by CRAs on-site via a RedCap (Research Electronic Data Capture) interface hosted at Pasteur Institute of Madagascar (IPM) [29–31]. Data was entered a second time at IPM in Antananarivo. Routine data checks ensured that errors, discrepant values, and missing data could be addressed. Data entry was supervised by the data management team and the study's medical coordinator.

#### Data analysis

The primary endpoint of the study was the change in the prevalence of malaria RDT positivity in the intervention *versus* control *fokontany*, analyzed using a difference-in-differences (DiD) approach comparing baseline to endline.

First, descriptive analyses of individual and household characteristics for the baseline and endline surveys were performed. To identify individual and household factors associated with RDT positivity, a mixed-effects logistic regression model that accounted for clustering via a random intercept was used for the baseline and endline populations. Models included study arm, demographic characteristics (e.g., age, sex), highest education level attained among household members, and prevention behaviors (e.g., sleeping under an LLIN) identified through the baseline questionnaire.

Key process indicators were estimated in the intervention arm. These included the average by *fokontany* and each round of pro-CCM visits (every 2 weeks) of percent of households visited out of the households registered in the initial census, percent of households that were visited and gave consent for the screening, fever and malaria incidence out of all individuals screened, and CHW adherence to study protocols, namely the percent of fever cases with RDT performed, and the percent of RDT-positive individuals treated with an ACT.

A DiD analysis of malaria prevalence between the two arms at baseline and endline was done using a logistic regression model of individual RDT positivity. To account for clustering, we used generalized estimating equations with an exchangeable correlation matrix and robust standard errors. Since IRS can have a substantial impact on reducing malaria prevalence which could bias the study results, analyses were adjusted for IRS implementation. The following model was used:

1 $$Y=\alpha +{\beta}_1\ period+{\beta}_2 arm+{\beta}_3\ \left( period\times arm\right)+{\beta}_4 IRS+{\beta}_5\ \left( period\times IRS\right)+\varepsilon$$

In this model, *β*_1_ represents the average difference in RDT positivity between endline and baseline; *β*_2_ accounts for differences in the prevalence of positive RDTs at baseline by arm; and *β*_3_ represents the DiD estimator of the intervention impact, namely the interaction between time and arm. Given the potential impact of IRS on malaria prevalence, for the 10 *fokontany* having benefited from IRS (4 in the intervention and 6 in the control arms), the model accounted for baseline differences in malaria prevalence between *fokontany* by IRS status (*β*_4_) and for the impact of IRS over time (*β*_5_). The variables *α* and *ε,* are the intercept and error, respectively.

Intention-to-treat and per-protocol analyses were performed. Intention-to-treat analyses were performed using the entire study population, to compare the prevalence of positive RDTs at baseline and endline between the study arms and included all consenting individuals present at either the baseline or endline surveys. Per-protocol analyses were performed using only individuals who were present both at baseline and endline. Separate models were constructed for individuals of all ages and for the following age groups: under 5 years, 5–14 years, and ≥ 15 years. Given similar effects in models for children under 5 years and 5–14 years, we explored an additional model combining these two age groups. The models for individuals of all ages and for children under 15 years controlled for the individual’s age group in addition to variables described in Eq. 1. In-sample predictions of malaria prevalence from the final multivariate model for each age group were used to illustrate the absolute change in prevalence following implementation of pro-CCM and IRS. Analyses were carried out using R version 3.5.1 and R package “gee” [14]. Mapping was performed with QGIS version 2.18.

In addition to analyses of malaria prevalence, we compared anemia among women of reproductive age between intervention and control arms. Among non-pregnant women, no anemia was defined as hemoglobin (Hb) ≥ 12 g/dL, mild anemia as 11 ≤ Hb < 12 g/dL, moderate anemia as 8 ≤ Hb < 11 g/dL, and severe anemia as Hb < 8 g/dL. Among pregnant women, no anemia was defined as hemoglobin (Hb) ≥ 11 g/dL, mild anemia as 11 > Hb ≥ 10 g/dL, moderate anemia as 10 > Hb ≥ 7 g/dL, and severe anemia as Hb < 7 g/dL. We used a logistic regression model to perform a difference-in-differences (DiD) analysis, comparing none to any anemia and none or mild to moderate or severe anemia. We used a logistic regression model to examine differences in all anemia categories. Unadjusted analyses as well as adjusted analyses including RDT result, education level, LLIN use, parity, age < 20 years, and IRS in the cluster were conducted. We performed this analysis both for all women of reproductive age in both baseline and endline surveys (“intention-to-treat” analysis), and a sensitivity analysis for only women for whom we had both baseline and endline data (“per-protocol” analysis). As in analyses of malaria prevalence, generalized estimating equations were used to account for clustering at the cluster level using an exchangeable correlation matrix and robust standard errors.

Finally, we estimated average rates of fever, RDT screening, malaria confirmed cases, and ACT treatments during standard iCCM in each arm during pro-CCM implementation and in the 2 years prior for the subset of fokontany for which we had data, and we compared them with equivalent rates during pro-CCM in the intervention arm (Additional file 1: Fig. S3).

## Supplementary Information

**Additional file 1: Figure S1.** Flowchart of the households and individuals included on the survey from the baseline. **Figure S2.** Comparison of malaria incidence and positivity in the intervention area during March-October 2017 with average values for Mananjary District. **Figure S3.** Comparison of malaria community case management in the intervention and control arms during pro-CCM implementation and during the two years prior. **Figure S4.** Map of Mananjary district and the fokontany included in the intervention and control arms.

**Additional file 2. Table S1.** Distribution of positive RDT results in the 22 *fokontany.* **Table S2.** Impact of Pro-CCM and IRS on malaria prevalence, per-protocol analyses. **Table S3.** Comparison of observed proportion of parasite prevalence by RDT by age group between intervention and control arms. **Table S4.** Comparison of reported bed net use (LLIN) by age group between baseline and endline surveys.

**Additional file 3. **Analyses and findings on anemia and PRO-CCM. **Table S5.** Prevalence of anemia among all women of reproductive age at baseline and endline, by study arm. **Table S6.** Unadjusted analyses of prevalence of anemia among all women of reproductive age at baseline, using logistic regression models. **Table S7.** Adjusted analyses of prevalence of anemia among all women of reproductive age at baseline, using logistic regression models.

## Data Availability

The data described in this article can be openly accessed at Harvard Dataverse 2021: 10.7910/DVN/IIDE2B [32].

## Abbreviations

ACTs: Artemisinin-based combination therapies
CHW: Community health worker
CI: 95% confidence interval
CRAs: Clinical research assistants
CU5: Children under 5 years
DiD: Difference-in-differences analysis
HCC: Health care center
iCCM: Integrated community case management
IRS: Indoor residual spraying
LLIN: Long-lasting insecticidal net
OR: Odds ratio
PCV: Peace Corps Volunteers
Pro-CCM: Proactive community case management
RDT: Rapid diagnostic test
SSA: Sub-Saharan Africa
WHO: World Health Organization
