# Supplementary material for: Proactive community case management decreased malaria prevalence in rural Madagascar: results from a cluster randomized trial
Source: BMC Med. 2022 Oct 4;20:322. doi: 10.1186/s12916-022-02530-x (PMC9531497; doi:10.1186/s12916-022-02530-x)
Supplement: Supplementary file 1 — Additional file 1: Figure S1. Flowchart of the households and individuals included on the survey from the baseline. Figure S2. Comparison of malaria incidence and positivity in the intervention area during March-October 2017 with average values for Mananjary District. Figure S3. Comparison of malaria community case management in the intervention and control arms during pro-CCM implementation and during the two years prior. Figure S4. Map of Mananjary district and the fokontany included in the intervention and control arms. [file 12916_2022_2530_MOESM1_ESM.docx]

**ADDITIONAL FILES 1: FIGURES S1 – S4**

Control:
3,056 households
13,366 individuals

Excluded:
Decline to participate: 16 households 1373 individuals

Total included :
**6,390 households and 27,292 individuals**

Intervention arm:
3,350 households
15,299 individuals

Assessed for eligibility:
 **6,406 households
28,665 individuals**

**BASELINE:**

**FOLLOW-UP:**
Mars – Oct 2017

Included in Intervention arm :
 May: 5 households 16 individuals
 June: 18 households 66 individuals
 July: 2 households 13 individuals
 Aug: 5 households 25 individuals
 Sept: 5 households 28 individuals
 Oct: 12 households 57 individuals

Intervention arm:
 3,342 households
14,469 individuals

Control arm:
 3,048 households
12,823 individuals

Intervention arm:
 3,295 households
14,264 individuals

Control arm:
 3,048 households
12,823 individuals

**Dec 2016 – Feb 2017:
6,343 households
27,087 individuals**

Flowchart (continuing) from the 6,406 households registered during the baseline

Not found in the endline data: 192 households

**ENDLINE:**November 2017 – January 2018
**Household data**

Intervention arm:
138 households

Control arm:
54 households

Intervention arm:
Consent not obtain (absents):
25 households

**Households identified : 6,214 households**

**Households contacted : 6,189 households**

Intervention arm:
3,212 households

Control arm:
3,002 households

Refusals
556 households

Refusals
 638 households

Intervention arm
3,187 households

Participants
2,364 households

Participants
2,631 households

Control arm
3,002 households

**Individual data**

**Participants :
4,995 households and 24,877 individuals**

Control arm
11,488 individuals

Intervention arm
13,389 individuals

Individuals not participants

Refusals: 555
Deceased: 42
Absents from the households: 1,086

Refusals: 794
Deceased: 75
Absents from the households: 1,850

**Individuals ‘participants:
20,475 individuals**

Control arm
**9,805 individuals**

Intervention arm **10,670 individuals**

Flowchart (continuing) from individuals presented during baseline and endline

**Individuals presented during baseline and endline:
17,879 individuals**

Individuals presented during baseline period (December 2016 – February 2017)

Control arm **8,648 individuals**

Intervention arm **9,231 individuals**

New arrivals presented during endline:
**2,596 individuals**

Intervention arm
46 individuals

From follow-up (Mar – Oct 2017)

Intervention arm
1,393 individuals

Control arm
1,157 individuals

New arrivals included during endline

**ADDITIONAL FILE 1. Figure S1. Flowchart of the households and individuals included on the survey from the baseline**


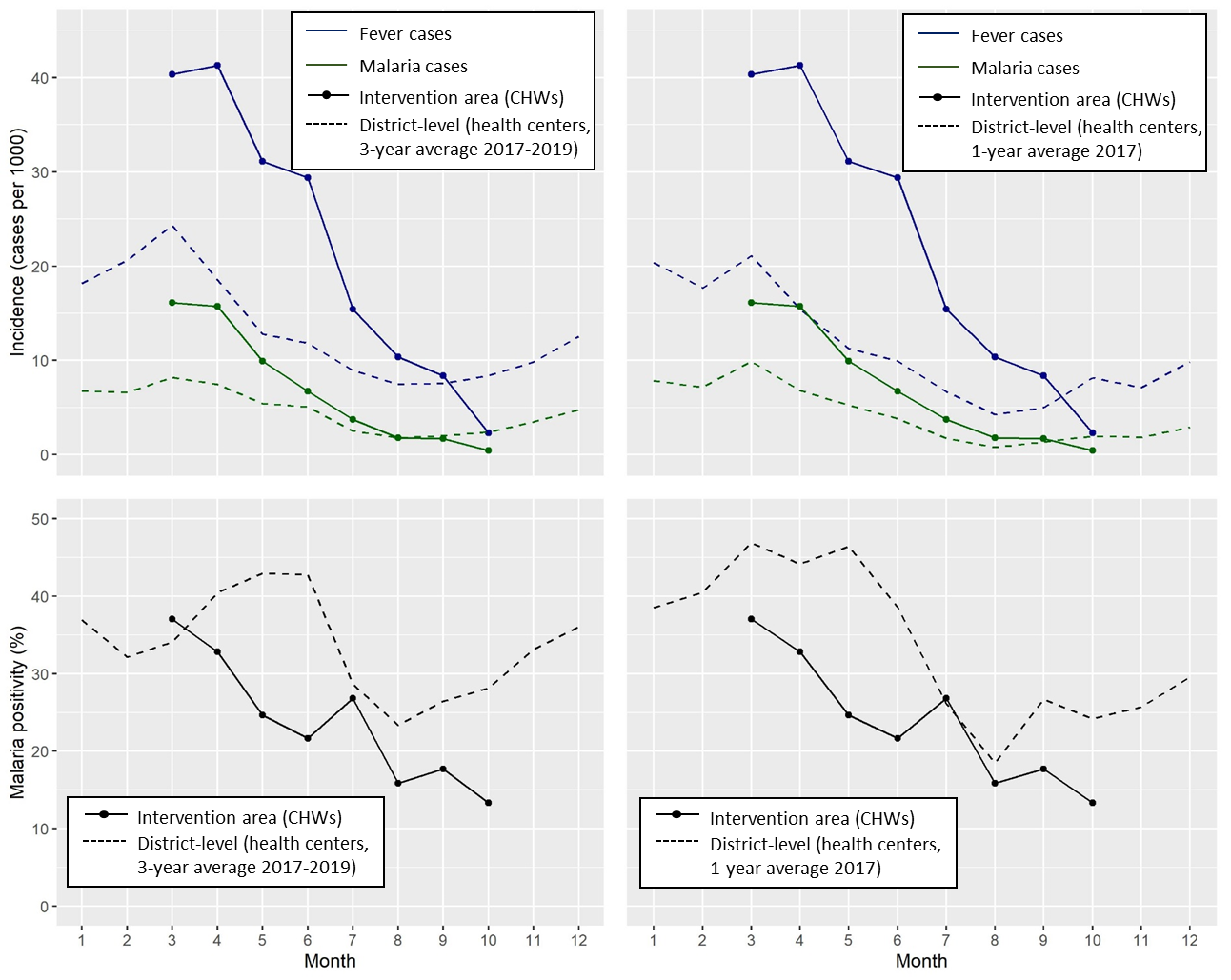


**ADDITIONAL FILE 1. Figure S2. Comparison of malaria incidence and positivity in the intervention area during March-October 2017 with average values for Mananjary District.** Solid lines show the evolution of average values per month for fokontany in the intervention arm, estimated from two pro-CCM visits (every two weeks). Dashed lines show 3-year (left) or 1-year (right) monthly average values for Mananjary (2017-2019) estimated from all 48 public and private health centers in the district. Colors represent different indicators. Please note that only one pro-CCM visit was completed in October 2017.

**
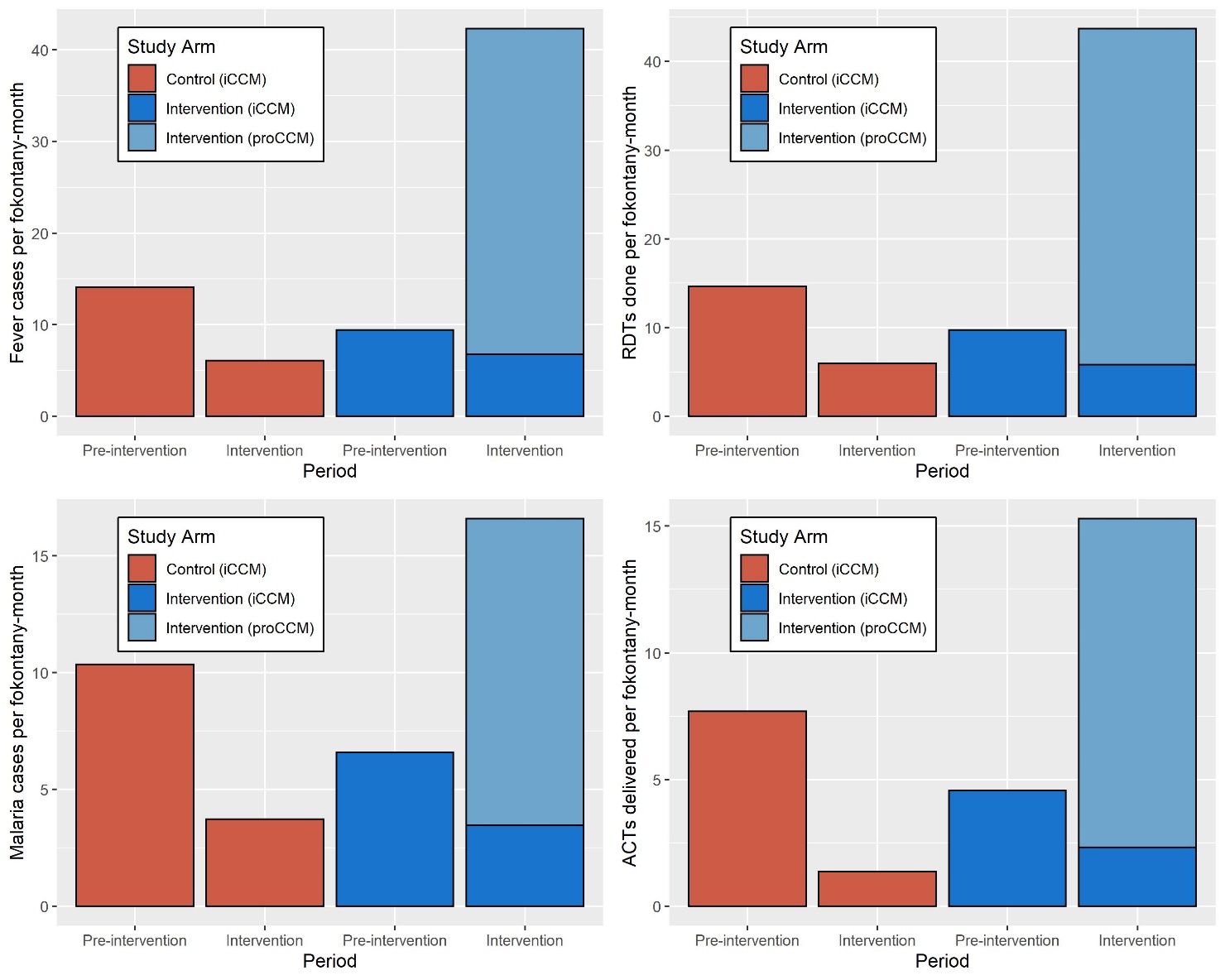
**

**ADDITIONAL FILE 1. Figure S3. Comparison of malaria community case management in the intervention and control arms during pro-CCM implementation and during the two years prior.** Bars show, for key indicators of malaria community case management, average values per month for fokontany in the intervention (blue) and control (red) arms, estimated from a subset of reports of community health workers during standard iCCM (passive screening) and from pro-CCM visits (active screening, light blue).


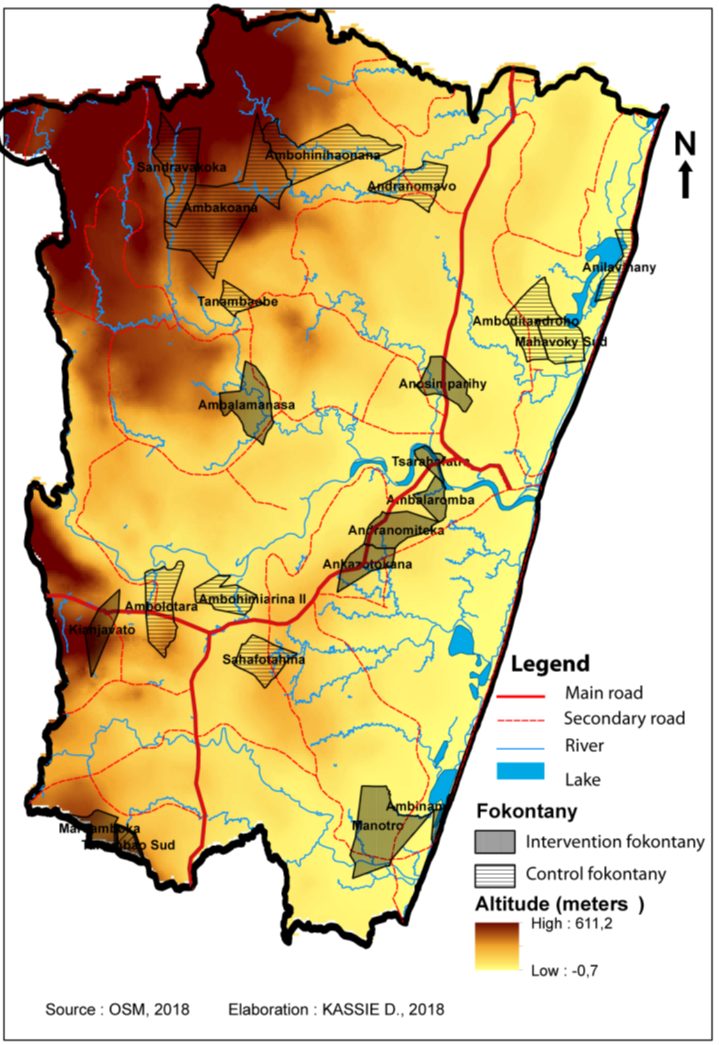


**ADDITIONAL FILE 1. Figure S4. Map of Mananjary district and the fokontany included in the intervention and control arms.** Map shows details on the spatial distribution of elevation, roads, rivers and lakes.
