# Supplementary material for: Proactive community case management decreased malaria prevalence in rural Madagascar: results from a cluster randomized trial
Source: BMC Med. 2022 Oct 4;20:322. doi: 10.1186/s12916-022-02530-x (PMC9531497; doi:10.1186/s12916-022-02530-x)
Supplement: Supplementary file 2 — Additional file 2. Table S1. Distribution of positive RDT results in the 22 fokontany. Table S2. Impact of Pro-CCM and IRS on malaria prevalence, per-protocol analyses. Table S3. Comparison of observed proportion of parasite prevalence by RDT by age group between intervention and control arms. Table S4. Comparison of reported bed net use (LLIN) by age group between baseline and endline surveys. [file 12916_2022_2530_MOESM2_ESM.docx]

**ADDITIONAL FILES 2: TABLES S1 – S3**

**ADDITIONAL FILE 2. Table S1.** Distribution of positive RDT results in the 22 *fokontany*, for the intervention and control arms, at baseline and endline.

| **Intervention arm (11 fokontany)** | | | | | **Control arm (11 fokontany)** | | | | |
| --- | --- | --- | --- | --- | --- | --- | --- | --- | --- |
| Names of the fokontany | Prevalence of positive RDT | | | | Names of the fokontany | Prevalence of positive RDT | | | |
|  | BASELINE  n/N *%* | | ENDLINE  n/N *%* | |  | BASELINE  n/N *%* | | ENDLINE  n/N *%* | |
| Ankazotokana | 224/1,315 | *17.0* | 159/1,109 | *14.3* | Ambohimiarina II* | 87/1,246 | *7.0* | 35/690 | *5.1* |
| Kianjavato | 18/1,614 | *1.1* | 4/861 | *0.5* | Sahafotahina | 64/1,105 | *5.8* | 64/817 | *7.8* |
| Ambinany Namorona* | 120/1,060 | *11.3* | 63/918 | *6.9* | Ambolotara | 31/1,038 | *3.0* | 18/554 | *3.2* |
| Manotro* | 273/1,516 | *18.0* | 133/1,002 | *13.3* | Anilavinany* | 40/1,103 | *3.6* | 22/925 | *2.4* |
| Andranomiteka | 214/1,099 | *19.5* | 49/557 | *8.8* | Ambohinihaonana* | 147/1,310 | *11.2* | 100/1,181 | *8.5* |
| Tsarahafatra | 19/1,326 | *1.4* | 18/1,022 | *1.8* | Andranomavo* | 100/1,567 | *6.4* | 46/1,048 | *4.4* |
| Ambalaromba | 48/1,331 | *3.6* | 11/1,028 | *1.1* | Mahavoky Sud* | 117/1,142 | *10.2* | 36/1,086 | *3.3* |
| Anosimparihy | 58/1,180 | *4.9* | 20/1,062 | *1.9* | Amboditandroho* | 99/1,094 | *9.0* | 31/768 | *4.0* |
| Ambalamanasa | 24/1,464 | *1.6* | 16/1,096 | *1.5* | Ambakoana | 60/1,022 | *5.9* | 84/941 | *8.9* |
| Tanambao Sud* | 100/1,195 | *8.4* | 77/1,067 | *7.2* | Sandravakoka | 90/1,090 | *8.3* | 91/739 | *12.3* |
| Maroamboka* | 43/1,164 | *3.7* | 24/948 | *2.5* | Tanambaobe | 40/1,106 | *3.6* | 33/1,056 | *3.1* |
| Total | 1,141/14,264 | *8.0* | 574/10,670 | *5.4* | Total | 875/12,823 | *6.8* | 560/9,805 | *5.7* |

* *Fokontany with IRS*

**ADDITIONAL FILE 2. Table S2.** Impact of Pro-CCM and IRS on malaria prevalence, per-protocol analyses (multivariate results, generalized estimating equations)

| **Variable** | **Individuals all ages^1^** | **Children less than 15 years^2^** | **Children under 5 years** | **Children 5 to 14 years** | **Individuals 15+ years** |
| --- | --- | --- | --- | --- | --- |
|  | **OR (95% CI)** | **OR (95% CI)** | **OR (95% CI)** | **OR (95% CI)** | **OR (95% CI)** |
| Intercept | 0.05  (0.03-0.08)*** | 0.15  (0.03-0.47)* | 0.03  (0.01-0.06)*** | 0.14  (0.09-0.21)*** | 0.03  (0.02-0.05)*** |
| **Differences at baseline** |  |  |  |  |  |
| Between arms (intervention vs control) | 0.99 (0.54-1.82) | 0.26 (0.02-3.47) | 1.83 (0.92-3.65) | 0.91 (0.49-1.68) | 1.11 (0.59-2.06) |
| Between IRS status (receiving vs. not) | 0.96 (0.5-1.83) | 0.26 (0.02-3.61) | 1.34 (0.57-3.13) | 0.9 (0.46-1.74) | 1.37 (0.74-2.52) |
| **Differences over time** |  |  |  |  |  |
| End line vs baseline | 1.02 (0.7-1.47) | 0.96 (0.77-1.19) | 1.06 (0.49-2.29) | 0.9 (0.66-1.23) | 1.2 (0.76-1.89) |
| **Impact of interventions** |  |  |  |  |  |
| Impact of Pro-CCM over time (DiD) | 0.73 (0.47-1.14) | 0.63 (0.38-1.05) | 0.71 (0.32-1.59) | 0.7 (0.46-1.06) | 0.83 (0.5-1.37) |
| Impact of IRS over time (DiD) | 0.71 (0.45-1.12) | 0.69 (0.45-1.05) | 0.91 (0.39-2.1) | 0.71 (0.48-1.04) | 0.68 (0.42-1.11) |

*p-value < 0.05; ** p-value < 0.01; *** p-value < 0.001

^1^ Model adjusted for age group; Children 0-4 years (ref); Children 5-14 years OR 2.49 (95% CI 1.82-3.39)***; Individuals 15+ years OR 0.72 (95% CI 0.5-1.04)

^2^ Model adjusted for age group; Children 0-4 years (ref); Children 5-14 years OR 2.38 (95% CI 2-2.83)***

**ADDITIONAL FILE 2. Table S3.** Comparison of observed proportion of parasite prevalence by RDT by age group between intervention and control arms.

| **Age group** | **Intervention arm** | | **Control arm** | |
| --- | --- | --- | --- | --- |
|  | **Baseline** | **Endline** | **Baseline** | **Endline** |
| Children under 5 years | 8.0% | 4.9% | 4.6% | 3.8% |
| Children 5 to 14 years | 14.6% | 8.9% | 12.6% | 10.2% |
| Individuals 15 years and older | 4.2% | 3.5% | 4.3% | 3.8% |

**ADDITIONAL FILE 2. Table S4.** Comparison of reported bed net use (LLIN) by age group between baseline and endline surveys.

| **Age group (years)** | **Baseline** | | **Endline** | |
| --- | --- | --- | --- | --- |
|  | **n** | **%** | **n** | ***%*** |
| 0-4 | 5257 | 92.1 | 3960 | 94.9 |
| 5-14 | 7998 | 87.9 | 6183 | 90.6 |
| 15-49 | 11447 | 89.3 | 8417 | 92.9 |
| >50 | 2385 | 91.7 | 1915 | 95.1 |
