# Supplementary material for: Proactive community case management decreased malaria prevalence in rural Madagascar: results from a cluster randomized trial
Source: BMC Med. 2022 Oct 4;20:322. doi: 10.1186/s12916-022-02530-x (PMC9531497; doi:10.1186/s12916-022-02530-x)
Supplement: Supplementary file 3 — Additional file 3. Analyses and findings on anemia and PRO-CCM. Table S5. Prevalence of anemia among all women of reproductive age at baseline and endline, by study arm. Table S6. Unadjusted analyses of prevalence of anemia among all women of reproductive age at baseline, using logistic regression models. Table S7. Adjusted analyses of prevalence of anemia among all women of reproductive age at baseline, using logistic regression models. [file 12916_2022_2530_MOESM3_ESM.docx]

**ADDITIONAL FILE 3. ANALYSES AND FINDINGS ON ANEMIA AND PRO-CCM**

A secondary objective of the study was to measure the impact of the intervention on the prevalence of anemia among women of reproductive age. For this, women aged 15 to 49 years were screened by hemoglobinometer (HemoCue Hb 201+) and received care if anemia was detected. Anemia prevalence was compared between intervention and control arms at baseline and endline. Among non-pregnant women, no anemia was defined as hemoglobin (Hb) ≥ 12 g/dL, mild anemia as 11 ≤ Hb < 12 g/dL, moderate anemia as 8 ≤ Hb < 11 g/dL, and severe anemia as Hb < 8 g/dL. Among pregnant women, no anemia was defined as hemoglobin (Hb) ≥ 11 g/dL, mild anemia as 10 ≤ Hb < 11 g/dL, moderate anemia as 7 ≤ Hb < 10 g/dL, and severe anemia as Hb < 7 g/dL. We used generalized estimating equations (GEE) and a logistic regression model to perform difference-in-differences (DiD) analysis, comparing none to any anemia, and none or mild to moderate or severe anemia. Unadjusted analyses as well as analyses adjusted for RDT result, education level, ITN use, parity, age < 20 years, and IRS in the cluster were carried out. Similar to the analysis of malaria prevalence in the main text, we performed this analysis both for all women of reproductive age in both baseline and endline surveys (“intention-to-treat” analysis), and a sensitivity analysis for only women for whom we had both baseline and endline data (“per-protocol” analysis).

At baseline, 57.8% (95% CI 55.4-64.2) of women of reproductive age had anemia in the control arm versus 61.1% (95% CI 44.5-66.7) in the intervention arm; at endline, 49.7% (95% CI 43.0-56.4) had anemia in the control arm versus 50.9% (95% CI 42.9-58.9) in the intervention arm (Table S5). The unadjusted difference-in-difference analysis showed a significant 10% decrease in anemia across both arms from baseline to endline (p=0.0015), but no difference over time by arm (DiD -0.13%, 95% CI -8.32-8.06) (Table S6). Results did not differ significantly between arms when no or mild anemia was compared to moderate or severe anemia (Table S6). In the adjusted analysis comparing none to any anemia, positive RDT (p< 0.0001), baseline (vs. endline) (p=0.0008), having elementary (p=0.0353) or secondary education versus none (p=0.0110), not sleeping under a bednet (p=0.0023), and age < 20 years (p=0.059) were positively associated with any anemia; in the adjusted analysis comparing none or mild anemia to moderate or severe anemia, positive RDT (p<0.0001), baseline (vs. endline) (p= 0.0013), having secondary, versus no, education (p=0.0017), not sleeping under a bednet (p=0.0027), and low gravidity (p= 0.008) were positively associated with moderate or severe anemia (Table S7). The DiD estimator was not significantly associated with either none compared to any anemia (p= 0.81) or none or mild compared to moderate or severe anemia (p=0.81).

**ADDITIONAL FILE 3. Table S5.** Prevalence of anemia among all women of reproductive age at baseline and endline, by study arm

|  | **Baseline**  **Control** | **Baseline Intervention** | **Endline**  **Control** | **Endline**  **Intervention** |
| --- | --- | --- | --- | --- |
| No anemia (Hb ≥ 12 g/dL in non-pregnant, Hb ≥ 11 g/dL in pregnant) | 40.1%  (35.4-44.9) | 38.9%  (32.8-45.0) | 51.1%  (43.9-58.3) | 48.7%  (39.8-57.5) |
| Mild anemia (12 > Hb ≥ 11 g/dL in non-pregnant, 11 > Hb ≥ 10 g/dL in pregnant) | 24.6%  (22.9-26.2) | 25.9%  (23.5-28.3) | 23.2%  (20.6-25.9) | 24.7%  (21.5-27.9) |
| Moderate anemia (11 > Hb ≥ 8 g/dL in non-pregnant, 10 > Hb ≥ 7 g/dL in pregnant) | 31.5%  (28.3-34.7) | 31.2%  (26.0-36.5) | 22.9%  (18.1-27.8) | 23.6%  (17.8-29.5) |
| Severe anemia (Hb < 8 g/dL in non-pregnant, Hb < 7 g/dL in pregnant | 3.7%  (2.4-4.9) | 3.9%  (0.7-7.2) | 2.7%  (1.4-4.1) | 3.0%  (0.0-7.2) |
| Any anemia (Hb ≥ 12 g/dL in non-pregnant, Hb ≥ 11 g/dL in pregnant) | 59.8%  (55.4-64.2) | 61.1%  (55.5-66.7) | 49.7%  (43.0-56.4) | 50.87%  (42.9-58.9) |
| Moderate to severe anemia (Hb > 11 g/dL in non-pregnant, Hb > 10 g/dL in pregnant) | 35.2%  (31.9-38.6) | 35.2%  (28.4-42.0) | 26.2%  (20.9-31.5) | 26.0%  (18.0-34.0) |

**ADDITIONAL FILE 3. Table S6.** Unadjusted analyses of prevalence of anemia among all women of reproductive age at baseline, using logistic regression models

|  | **Outcome: any anemia** | | | | **Outcome: moderate/severe anemia** | | | |
| --- | --- | --- | --- | --- | --- | --- | --- | --- |
|  | Odds ratio | 95% CI | | p-value | Odds ratio | 95% CI | | p-value |
| *All women (N=10,815)* |  |  |  |  |  |  |  |  |
| Time (endline vs. baseline) | 0.664 | (0.516, | 0.855) | 0.0015 | 0.654 | (0.501, | 0.855) | 0.0019 |
| Intervention | 1.056 | (0.783, | 1.424) | 0.7213 | 0.999 | (0.716, | 1.394) | 0.9962 |
| Intervention*Time [Did estimator] | 0.993 | (0.714, | 1.379) | 0.9642 | 0.990 | (0.680, | 1.442) | 0.9595 |

**ADDITIONAL FILE 3. Table S7.** Adjusted analyses of prevalence of anemia among all women of reproductive age at baseline, using logistic regression models
